# Notes and News

**Published:** 1889-09-14

**Authors:** 


### Notes and News.

Collected and Communicated.

Religion is not forgotten at the Seamen's Hospital. During the year there have been morning services every weekday in the chapel, as well as morning and evening services on Sundays, and 26 communion services. The attendances throughout the year have been good. Every ward has been visited by the chaplain once a week, and urgent cases daily.

Melbourne is just now excited over the hospital question. A new building in a healthy site is necessary for the sick. We should advise Melbourne to amalgamate the General and Victoria Hospitals into one strong efficient institution. The evils of multiplying small hospitals become more apparent daily in England, and the same causes are at work in the colonies.

On Tuesday, the 3rd inst., a very successful concert was given at the Torbay Hospital; £20 was handed over to the funds. The whole expenses were only £2. The nurses showed the visitors to their seats, attended to the cloak room, and sold programmes. It is hoped next year to give a larger entertainment for the same cause in the Bath Saloon, the ward being too small.

Hastings Hospital, opened in October, 1887, still has a debt of £3,000 on the building fund. The total number of patients in 1888 was 2,639, being an increase of 623 as compared with the previous year. Of this 2,639, 416 were in-patients, and 2,223 out-patients. Of the cases admitted last year, 140 belonged to the special department—namely, 55 diseases of women, 32 eye cases, and 53 ear affections.

It is as well to compare figures at times to prevent their being very misleading. When Sir H. Roscoe stated at the Mansion House that 214 persons bitten by rabid dogs had been sent to Pasteur from England, and the mortality had only been 3·7 per cent., the statement was received with cheers. But in the *Lancet* for June 15, we learn that 307 similar cases have been registered at Katherine Hospital and the mortality was only 2·6 per cent.

In his last volume of poems, Oliver Wendell Holmes turns frequently to medical subjects for a theme. There is a hymn read at the dedication of the Oliver Wendell Holmes Hospital at Hudson, Wisconsin, in 1887; there is a long description of the pleasures of a morning visit from a cheerful physician who has acquired the proper sick-room manner, and in the "Death of Garfield," and "Before the Curfew," are phrases and similes which betray the medical man. Here is a specimen:

> "Calm as a clock your knowing hand directs,

> Rhei, jalapæ ana grana sex,

> Or traces on some tender missive's back,

> Scrupulos duos pulveris ipecac."

Hospital patients are not always grateful and polite to the authorities, still it seems strange that one should have so little consideration for his fellow-sufferers as to set fire to an infirmary. Yet this occurred in Switzerland, at Muri, last week, and the building was almost wholly destroyed. The patient has been arrested, and, it is stated, is a man of means.

Here is news of a threatened new hospital in London. An anonymous lady has promised £1,000 towards an Anti-Vivisection Hospital. We confess we scarcely know what will be the speciality of this hospital, unless it be that Burdon Sanderson and Lander Brunton will never be allowed within its portals. Hospitals in general are not hot-beds of vivisection, whatever the screaming fraternity may aver.

The particulars of the leper colony at Robben Island, given by a contributor to *Blackwood*, are almost too realistic. On landing he found himself in the midst of a settlement of about 550 souls—30 of them convicts, 230 lunatics, 130 lepers, and the rest police and ward-masters with their families. There is a hospital, and here, in bare, miserable, tumble-down wards, often with the ground for floor, crouch the victims of the disease, most of them belonging to a race which is partly Hottentot and partly Malay. There is no murmuring of small talk, no muttering of suffering; an unbroken silence is the lepers' cry. Two patients are thus described: "He is quite helpless and in bed, but the doctor uncovers him sufficiently to show the ravages. Legs, or rather what remains of them, drawn up, and their extremities resembling round rulers. Arms in a far worse condition—they are now scarcely longer than from an ordinary shoulder to elbow distance; in fact, they are perfectly useless, shapeless stumps, with rudimentary fingers, but perfect finger-nails projecting from a small knob at the base of the stump, which once represented a hand. Truly here is absorption of a man's whole corporeal being; his very collar-bones and shoulder-blades seem to be in process of rapid diminution; he is undergoing a living death." Of the second patient it is said: "Probably he is not much over thirty, but his malady has reached even his arms, which are pulpy and inflated, and the skin resembles the rough rind of a boiled orange. His ear lobes are dangling down in grotesque lumps, huge knobs are disposed about his cheeks, his nostrils are swollen monstrously, the nape of his neck is scarcely to be distinguished, and shocking, shocking beyond my powers of description, is a gigantic excrescence on the side of his head, which has disfigured him almost beyond recognition as a human being. His eyes are directed towards us with an expressionless fixity, his body is motionless, his whole being seems utterly torpid, he looks as though he were but half alive—would, indeed, that he were wholly dead!" The author says his reason for publishing these horrors is to try and find another Father Damien to take charge of the poor victims.

Whatever the fate of the lepers on Robben Island—and there is reason to believe their wretchedness has been exaggerated—it is not so horrible as that of a leper in England in olden times. When a man was accused of leprosy, he was examined by a doctor; and if found "guilty," all hope was gone, and he was compelled to bow to the terrible verdict of banishment from the society of his fellow men, which the law pronounced necessary for the health of the community. He was taken to a church, where a fearful and gloomy service was read over him. Often the mass for the dead was used, and earth was thrown upon his feet, the priest reciting such sentences as "Sis mortuus mundo, vivens autem Deo." Ten terrible prohibitions were laid upon him; and after a blessing from the priest, the poor wretch went forth into solitude. Thus the grave closed almost literally over him.

The following vacancies are announced this week, the amount of the salary in each case being given after the name of the institution:

**Resident Medical Officers.**

Bethlem Hospital. Two Resident Clinical Assistants.—Board, no salary.
Chester General Infirmary. Visiting Surgeon.—£80, with board, &c.
City of London Hospital for Diseases of the Chest, Victoria Park, E. House Physician.—Board, no salary.
City Asylum, Birmingham. Resident Clinical Assistant.—Board, no salary.
County Asylum, Shrewsbury. Junior Assistant Medical Officer.—£108, board, &c.
Great Northern Central Hospital, Holloway-road, N. House Surgeon.—£80, board, &c.
Ipswich Borough Lunatic Asylum. Resident Medical Officer.—£400, with unfurnished house.
Liverpool Infirmary for Children. Assistant House Surgeon.—Board, no salary.
Macclesfield General Infirmary. Senior House Surgeon.—£100, board, &c.
Poplar Hospital for Accidents, E. Assistant House Surgeon.—£50, board, &c.
Royal Albert Hospital, Devonport. Resident Medical Officer.—£150, board, &c.
Royal National Hospital for Consumption, Ventnor. Assistant Medical Officer.—£60, board, &c.
Royal South Hants Infirmary. House Surgeon.—£100, board, &c.
Scarborough Hospital and Dispensary. House Surgeon.—£80, board, &c.
West London Hospital, Hammersmith-road, W. House Surgeon.—Board, no salary.

**Non-Resident Medical Officers.**

Basford Union Sanitary Authority. Medical Officer of Health.—£400.
Luton Friendly Societies' Medical Institute. Assistant Medical Officer.—£120.
Glasgow Royal Infirmary and St. Mungo's College. Curator of Museum.—£50.

---

Amongst the many congresses which have been at work lately was an anti-vaccination one, under the presidency of Dr. Hubert Boens, of Belgium. Dr. Oidemann, of Aix-la-Chapelle, gave some interesting information in a paper on the present state of the question in Germany. Till lately it was customary in that country, and indeed at an earlier date nearly all over Europe, to vaccinate healthy sheep with the virus from diseased animals. This is now not only not compulsory, but positively illegal. On the other hand, vaccination of children is compulsory. Dr. Oidemann thinks that public opinion in Germany is in favour of making vaccination optional, with certain restrictions as to the consent of parents or relatives.

---

At the recent meeting of the British Medical Association, the question of disinfectants occupied a large portion of the attention of one of the sectional gatherings. The medical men present, by general consent, declared that the two principal methods of preventing the spread of zymotic and infectious diseases of all kinds were the free use of disinfectants and thorough isolation of cases. Whilst disinfectants should not be relied upon to take the place of cleanliness, there is no doubt of the extreme value of applications of this kind to drains and other places in which putrefaction takes place, and in which thorough lustration and cleansing are impossible. There are many reliable disinfecting substances in the market. Indeed, so keen is the competition among manufacturers, that articles which are not reliable cannot hope to continue to command a sale. Among the many different varieties of disinfectants that have been sent to us none holds a higher place than Jeyes' "perfect purifiers." They are put up in all kinds of convenient forms, and are always ready for use. We have no hesitation in commending them warmly to the attention of our readers.

The annual report of Horton Infirmary for Banbury and neighbourhood is now published. The tabular statement of the house surgeon shows that the number of cases treated in the infirmary during the past year was 310, whilst 295 accidents and urgent cases have been relieved, but not taken in as resident patients, making a total of 605 medical and surgical cases, to which must be added the dental cases reaching to the large number of 403, making a total of 1,008 persons. Funds are satisfactory, and there is no debt.

---

St. Mary's Hospital prides itself on the improvement of its out-patient maternity department, and on the successful start of its children's out-patient department. The need of this last department has long been felt, and its establishment is an important addition to the hospital, both as a charity for the relief of the sick poor and as a medical school. Mr. Edmund Owen, one of the surgeons of the hospital, and Dr. Sidney Phillips, one of the out-patient physicians, have been appointed surgeon and physician respectively.

---

The Sanitary Congress of Worcester will be divided into three sections: (1) Sanitary Science and Preventive Medicine; (2) Engineering and Architecture; (3) Chemistry, Meteorology, and Geology. There will be papers and discussions in each section, and addresses to the working classes. Earl Beauchamp will give a garden party to the members of the congress on September 25th. The Health Exhibition, including sanitary apparatus and appliances, in connection with the congress will be open to the public from September 24th to October 19th.

---

The last report of the National Hospital for the Paralysed and Epileptic contains a proposal for the allotment of beds to particular counties, towns, and districts, thus making the charity truly national. A list of 30 towns is given, and if one bed for a male patient and one for a female patient were provided and named by each of these towns, a very pleasing feature would be introduced into the wards of this national hospital; and, judging by the past, there would be more than a sufficiency of suitable applicants from these several places to keep the particular beds occupied.

---

On Saturday last the Duke of Cambridge laid the memorial stone of the Woolwich and Plumstead Cottage Hospital, a new institution, which stands at the foot of Shooter's Hill, and near the Herbert Hospital. Built of red brick, with Portland stone dressings, and of the Queen Anne style of architecture, the hospital presents a pretty appearance, and will, when completed, accommodate five male and five female patients, besides the matron and nurses. Mr. Woodford, churchwarden of St. Mary's, Woolwich, was the prime mover in its erection, the cost of the building alone being £2,700. Of this sum £500 was contributed by the War Office, the remainder being raised by voluntary subscriptions, and a further amount of £350 is still required to complete the structure. Some endowments are expected to be shortly made, and with an active worker like Mr. Woodford to support it, the hospital starts on the whole most favourably. The memorial stone bears the following inscription: "This hospital was erected to the glory of God and for the good of His poor. Also to commemorate the Jubilee year of the reign of Her Most Gracious Majesty Queen Victoria. This memorial stone was laid by His Royal Highness the Duke of Cambridge, K.G., etc., 7th Sept., 1889. Rev. S. G. Scott, chairman; Rev. Dr. Raitt, vice-chairman; Trustees: Rev. S. G. Scott, W. Woodford, J. J. Collins, W. Busbridge, E. W. Sampson; J. O. Cook, architect; R. Robson, treasurer; H. Coombes and A. White, builders; C. Edwards, sculptor."

